# MSX1 Mutation in Witkop Syndrome; A Case Report

**Published:** 2013-06

**Authors:** Faezeh Ghaderi, Somaye Hekmat, Reza Ghaderi, Majid Fardaei

**Affiliations:** 1 Department of Pediatric Dentistry, School of Dentistry, Shiraz University of Medical Sciences, Shiraz, Iran;; 2 Department of Dermatology, School of Medicine, Birjand University of Medical Sciences, Birjand, Iran;; 3 Department of Genetics, School of Medicine, Shiraz University of Medical Sciences, Shiraz, Iran

**Keywords:** Witkop syndrome, MSX1, Nail dysplasia

## Abstract

The Witkop syndrome is a rare autosomal dominant disorder characterized by the absence of several teeth and abnormalities of the nails. This is the first report of a rare genetic tooth and nail syndrome diagnosed in a 2.5-year-old boy with early exfoliation of the primary canine, absence of the primary incisors, and nail dysplasia. A homozygous mutation was identified in 3’-UTR of MSX1 gene in the proband. The parents of the patient had no dental and nail anomalies.

## Introduction

The Witkop syndrome, also known as the “tooth and nail syndrome” (TNS) or “nail dysgenesis and hypodontia”, is a form of ectodermal dysplasia, a group of hereditary diseases characterized by the absence or impaired function of two or more ectodermally derived structures such as teeth, hair, nails, and glands.^1^ Sweat glands and tolerance to heat are normal in the Witkop syndrome.^2^ This rare autosomal dominant disease was first reported by Witkop in 1965 and has a reported incidence of 1-2 in every 10000 born babies.^3^

The Witkop syndrome has certain characteristics. First and foremost among these characteristics is hypodontia, which is defined as morphological changes in teeth. Another feature is nail dysplasia: in this syndrome, nails tend to be spoon-shaped (koilonychia), thin, slow growing, and brittle (onychorrhexis) and toenails are generally affected more rigorously than fingernails. In some cases, the nail defects are improved with age and may not be obvious during adulthood.^1^^,^^2^^,^^4^ Permanent or primary teeth show different patterns of missing in the affected individuals and the alveolar bone is hypoplastic, leading to a lack of development of the jaw(s) and a reduced vertical dimension of occlusion. Lip eversion may occur due to the loss of occlusion in the vertical dimension. The residual teeth are usually markedly tapered, conical, or pointed.^2^ The gene responsible for the Witkop syndrome was discovered in 2001 and was named *MSX1*.^5^ *MSX1* is a transcription factor expressed in several embryonic structures, including the dental mesenchyme.^6^^,^^7^

In this study, we present the case of a 2.5-year-old boy with a mutation in *3’-UTR* of the *MSX1 *gene associated with the absence of the incisors, early exfoliation of the canines in primary dentition, and toe-nail dysplasia. Also in this study, we propose a simple Avall enzyme digestion for the analysis of this particular mutation.

## Case Description

**Figure 1:**
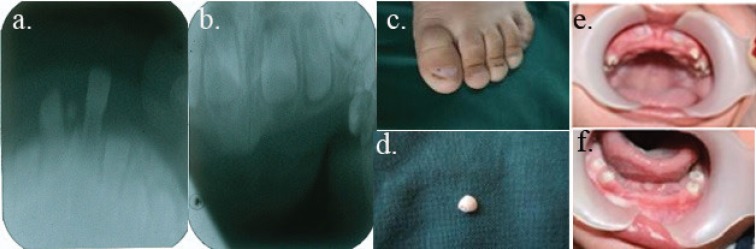
These are the clinical and radiographic manifestations of the patient’s condition. a. The mandibular anterior permanent germ in periapical view. b. The maxillary anterior permanent germ in periapical view. c. The child’s toenails are spoon–shaped and hypoplastic. d. The exfoliated mandibular primary canine. e and f. Clinical absence of all the maxillary primary incisors and mandibular anterior primary teeth, respectively.

**Figure 2:**
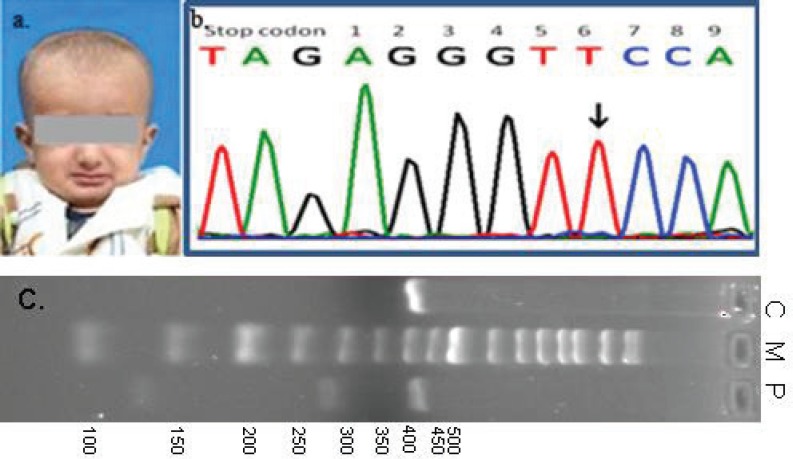
This is the child’s profile and MSX1 mutation. a. Child’ profile. b. DNA sequence of MSX1, including stop codon (TAG) and 9 nucleotides in 3’-UTR, is depicted. Homozygous 6C>T mutation in the DNA sequence of the patient is shown by arrow. c. PCR fragment of 413 bp in length was subjected to Avall digestion. While heterozygous control (C) was semi-digested into 413, 284, and 129 bp fragments, the homozygous mutant fragment (P) was not cleaved. The lane labeled M is a 50-bp ladder and its size is depicted on the left.

A 2.5-year-old boy was referred to the Dentistry Department of Pediatric Dentistry Faculty, Shiraz University of Medical Sciences, in June 2011. He presented with early exfoliation of the mandibular primary canines and maxillary right primary canine (figure 1d). In clinical examination, all the maxillary and mandibular primary incisors were missing (figures 1e-f). His parents stated that the primary incisors of their child had not erupted yet. Extraoral examination revealed lip eversion and fine hair, while the eyebrows and eyelashes were normal (figure 2a). No heat intolerance or any inability to sweat was reported. The toenails were spoon-shaped and hypoplastic (figure 1c).

Periapical radiography showed primary anterior germs (figures 1a-b). Panoramic view could not be taken due to the child’s poor cooperation. Regarding the early exfoliation of the primary canines, a diagnostic test was requested to determine the levels of serum alkaline phosphatase and urinary phosphoethanolamine, but no abnormality was reported. Oral examination of the patient’s parents revealed complete normal dentition and no abnormalities of the nails, scalp, hair, and eyebrows. There was no history of similar anomalies in the patient’s other family members except for a cleft palate in one of his maternal cousins. Genetic analysis was performed after obtaining written informed consent from the parents according to the ethical protocol of Shiraz University of Medical Sciences. DNA was isolated from peripheral blood leukocyte collected in EDTA via the standard salting out method. Two coding exons, exon-intron boundaries, and part of *3’-UTR *of* MSX1* were polymerase chain reaction (PCR) amplified, and the amplicons were subjected to mutation analysis by bidirectional direct sequencing (Bioneer, Korea). Amplification was performed for 3 minutes at 95^ο^C, followed by 35 cycles (30 seconds at 95^ο^C, 30 seconds at 59^ο^C, and 40 seconds at 72^ο^C) and 5 minutes at 72^ο^C. To avoid Taq polymerase-derived PCR errors, the PCR was carried out using Pfu DNA polymerase (Fermentas). Regarding the GenBank entry AF426432, one homozygous C>T variant, 6 nucleotides 3’ of the stop codon (*3’-UTR*) of *MSX1*, was identified (figure 2b). For a simple detection of this particular mutation, a restriction-enzyme analysis was also designed. Genomic DNA of this patient was amplified using X2.3F and X2.4R primers in a 50 μl PCR reaction.^8^ The PCR products were ethanol precipitated and dissolved in 10 μl of dH2O for digestion. The DNA products were subjected to Avall (Fermentas) enzyme digestion according to the recommendations of the manufacturer. The digested products were analyzed by 3% agarose gel electrophoresis. While control DNA with C/T genotype was semi-digested into three fragments, the patient’s sample (T/T genotype) was not digested by the Avall enzyme. At six months follow-up, the patient’s primary mandibular central teeth had erupted, but there was no eruption of the primary maxillary incisors and the left central tooth was lost. Due to the patient’s insufficient cooperation, occlusal view radiography was done to determine which permanent teeth germs were present. Unfortunately, permanent incisors teeth germs were not found in the radiographic view. 

## Discussion

The features of the Witkop syndrome are much less severe than those in usual ectodermal dysplasia, so it is likely to be missed by clinicians.^1^ In the present case, we focused on the chief complaint of the patient, which was early exfoliation of the primary canine teeth. To the best of our knowledge, this finding has not been reported in any case report of the Witkop syndrome yet. We were, therefore, suspicious of diseases that normally cause this early exfoliation of primary teeth such as hypophosphatasia, cyclic neuropenia, and Papillon-Lefèvre. Nevertheless, the results of blood and urine analysis-including alkaline phosphatase, phosphoethanolamine, calcium, and blood sugar, were normal. Moreover, the child did not present any sings such as palm and hand hyperkeratosis, which are usually seen in Papillon-Lefèvre, and nor did he have any compromising medical history such as recurrent infections. Igari et al*.*^9^ reported the case of a 5-year-old boy with premature exfoliation of the primary teeth. The patient had lost all of his primary incisors by the age of 3 and three primary canines and one primary first molar by the age of 4. Facial from, tapering of the finger, mental retardation, and motor dysfunction were seen in this case, which were inconsistent with the diagnosis of Coffin-Lowry syndrome.^9^ Our next differential diagnosis was the Witkop syndrome in light of the patient’s missing teeth, fine hair, toenail defects, and facial form. A few reported cases have fine or spare hair in addition to nail and teeth defects,^10^ and our patient had fine and spare hair. The most common missing teeth in the Witkop syndrome are maxillary incisors, canines, and the second molars.^10^ The affected teeth are conical and widely spaced and tend to have narrow crowns.^11^ Partial or total agenesis of permanent dentition is sometimes present and it subsequently results in over retention of the primary teeth,^12^ which is contrary to what we observed in our case (premature primary teeth exfoliation).

Considering the age of the patient and conspicuous developmental delay in some teeth such as incisors, one must wait to ascertain which teeth are missing. In a Witkop case presented by Altug-Atac AT et al.^13^ the absence of three mandibular incisors as well as spoon-shaped fingernails was reported. Jumlongras D et al.^5^ used candidate-gene linkage analysis in a three-generation family affected by the disorder to identify the gene responsible for the Witkop syndrome. The authors found an association between the appearance of the TNS and the presence of polymorphic markers which surround the *MSX1* locus. Finally, they identified a nonsense mutation in *MSX1* and concluded that this gene was critical for both tooth and nail development. Several other missense and nonsense mutations have also been reported in tooth agenesis patients.^14^^,^^15^ Homozygous deletion of *Msx1* in mice results in an arrest of tooth and nail development; this also supports the important function of the *MSX1* protein in tooth development.^5^ Compared to other findings, the mutation in the *3’-UTR *region in this study may explain the importance of post-transcriptional regulation or mRNA stability of *MSX1*. The exact functional consequences of this particular mutation should be investigated in further studies. The same variant identified in the present study was also previously reported for isolated clefting.^8^ An interesting aspect of the present case was early exfoliation of the primary canines and homozygous mutation in the *MSX1 *gene. The long variation in tooth germ development and eruption time was another observable aspect in this case. 

Despite our recommendations, the patient’s parents did not allow their child to wear prosthetics because they believed that he had posterior teeth and as such had no problems in chewing and mastication. Oral prescription of zinc-sulfate syrup was recommended to the patient as a complement medication to strengthen brittle nails. Some practitioners believe no treatment is usually required for nails,^16^ but hair oil is routinely proposed to reduce hair dryness in patients with this condition.^11^

## Conclusion

Clinicians should consider the possibility of the Witkop syndrome, although it is very rare, in their differential diagnosis when they face a patient with similar signs and symptoms.
